# Van Neck-Odelberg Disease — A Rare or Underdiagnosed Condition?

**DOI:** 10.1055/s-0041-1739402

**Published:** 2022-01-20

**Authors:** Mafalda Moreira, Diana Alba, Hélder Nogueira, Sandra Teixeira

**Affiliations:** 1 Serviço de Pediatria e Neonatologia, Centro Hospitalar do Tâmega e Sousa, Penafiel, Portugal

**Keywords:** child, intermittent claudication, osteochondrosis

## Abstract

Van Neck-Odelberg (VNO) disease is a rare osteochondrosis affecting the ischiopubic synchondrosis (IPS). This condition should be included in the differential diagnosis of children with lameness, inguinal pain, and functional limitation of the hip. In imaging tests, it is characterized by asymmetric IPS hypertrophy. We present the clinical case of a 4-year-old child, previously healthy, who visited the emergency department for left inguinal pain and lameness starting on the same day. There was no previous history of trauma or changes in inflammatory parameters. The patient underwent a pelvic radiography and magnetic resonance imaging (MRI), which revealed a radiopaque image with well-defined contours in the left ischiopubic branch, and IPS swelling. With a presumed diagnosis of VNO disease, the patient was medicated symptomatically, with complete recovery in 10 days. Lameness is a frequent reason for medical evaluation in the pediatric population. In subjects up to 5 years old, the most common causes of lameness include transient hip synovitis, septic arthritis, and Legg-Calvé-Perthes disease. In the absence of a history of trauma or infection-related clinical findings, VNO disease should be considered as a hypothesis. Its diagnosis requires a pelvic radiography, usually showing a unilateral fusiform opacification at the ischial level; an MRI may be necessary. The recommended treatment is conservative, with symptomatic recovery in 2 weeks. The knowledge and diagnosis of VNO disease allow a targeted approach, without the emotional burden for the patient and his/her family that may be associated to other conditions.

## Introduction

Osteochondrosis, also called osteochondritis by some authors, results from changes in endochondral ossification at the level of developing bone nuclei at epiphyses and apophyses. 1 2 Although its etiology remains unknown, vascular and microtraumatic changes seem to be play a role. 2 Osteochondrosis predominates in childhood and adolescence, and it is associated with growth spurts and hormonal changes. The most frequent osteochondroses include Köhler disease, Freiberg disease, Sever disease, and Osgood-Schlatter disease; in contrast, ischiopubic osteochondritis, or Van Neck-Odelberg (VNO) disease, is a rarely diagnosed osteochondrosis. 1

The first description of ischiopubic osteochondritis was made in 1923 by Van Neck, and later complemented by Odelberg in 1924; as such, it is known today as Van Neck-Odelberg disease. 3 4 It is a benign, self-limiting condition characterized by imaging evidence of asymmetric ischiopubic synchondrosis (IPS) hypertrophy, associated with lameness, unilateral inguinal pain, and functional limitation of the hip. 3 4 The differential diagnoses list include stress fracture, infectious conditions (i.e., osteomyelitis), or neoplastic diseases; some cases require magnetic resonance imaging (MRI) for diagnosis. 3 5 6

## Case Report

A 4-year-old girl, otherwise healthy, visited the emergency department with lameness and left inguinal pain for 24 hours, with progressive worsening. She presented no fever, constitutional symptoms, or local inflammatory signs. There was no previous history of trauma or recent infectious complications. An objective examination revealed pain during hip mobilization, with no local inflammatory signs.

**Fig. 1:**
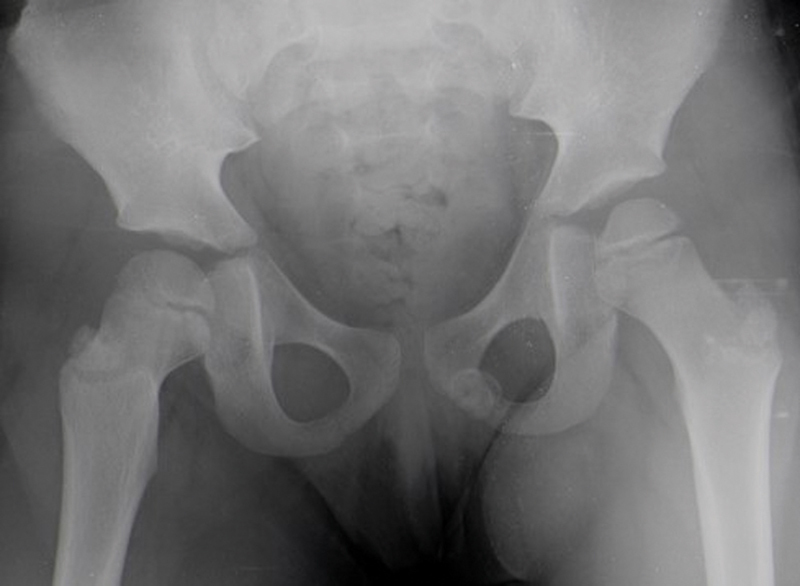
Radiography of the pelvis (anteroposterior view), revealing a radiopaque image at the level of the left ischiopubic branch, with well-defined contours.

**Fig. 2:**
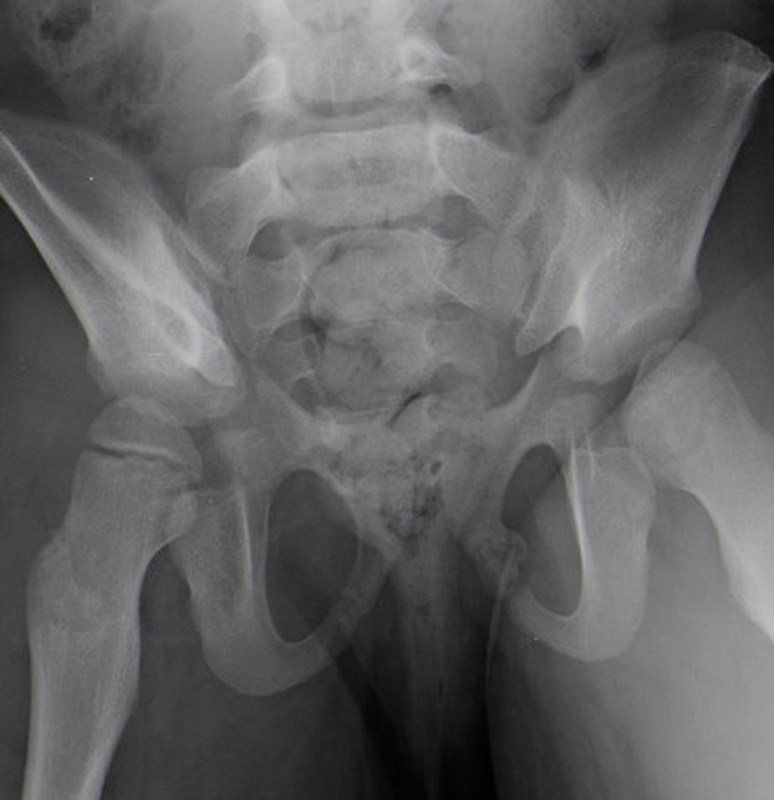
Radiography of the pelvis (outlet view).

**Fig. 3:**
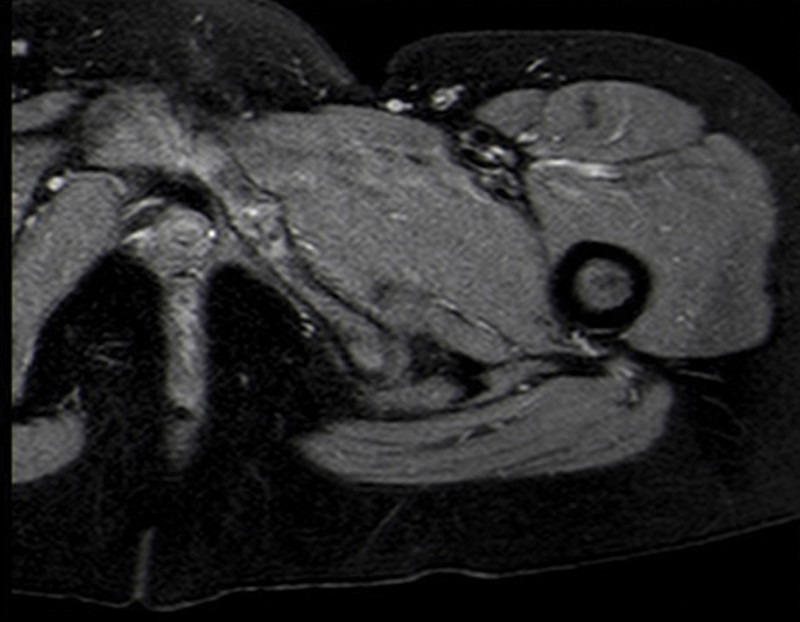
Magnetic resonance imaging of the pelvis, revealing edema at the level of the left ischiopubic synchondrosis.

An analytical study, including complete blood count, erythrocyte sedimentation rate (ESR), and C-reactive protein (CRP), revealed no major changes. A pelvic radiography showed a radiopaque image, about 1.8 × 1.4 cm in size, with well-defined contours, at the level of the left ischiopubic branch ( Figs. 1 and 2 ). For etiological clarification, an MRI of the pelvis was performed, which showed edema at the level of the left ischiopubic synchondrosis, with no abnormalities in the adjacent soft tissues ( Fig. 3 ).

Excluding the hypotheses of infectious or tumoral etiology, a diagnosis of VNO disease was presumed. The patient was symptomatically medicated with oral antiinflammatory and rest. Ten days later, she was reassessed clinically, with no complaints.

**Fig. 4:**
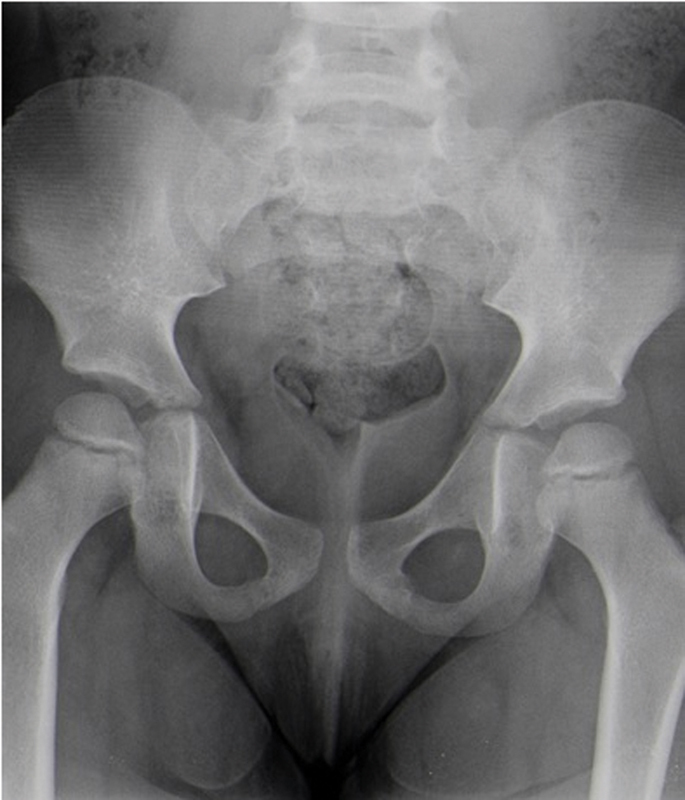
Radiography of the pelvis (anteroposterior view), revealing complete recovery.

A radiograph of the pelvis was repeated 6 months later and showed complete remodeling of the lesion area ( Figure 4 ). The patient presented no new episodes.

## Discussion

Lameness is a frequent complaint in the pediatric population. Its differential diagnosis is very challenging due to the multiple potential etiologies, which vary according to the age group. In children with unilateral inguinal pain and limited hip mobility but no fever, history of trauma, or recent infections, VNO disease is an important consideration. 6

This condition results from the asymmetric ossification process of the IPS, which usually begins in childhood and ends before adolescence. 5 In most cases, it does not cause symptoms. However, some children may present inguinal or gluteal pain and joint mobility limitation.

Van Neck-Odelberg disease is usually more frequent in the non-dominant lower limb, mostly the left one, as in the case of our patient. Some authors believe that this is due to body weight support mainly by the non-dominant lower limb compared to the contralateral one, which is used preferentially for movement execution. 3 5 This force imbalance may delay the IPS closing at the non-dominant side, which, due to mechanical stress, may become painful. 3 5

Van Neck-Odelberg osteochondritis is a diagnosis of exclusion in children with lameness. 6 Its diagnosis requires a pelvic radiography, which often reveals a unilateral fusiform opacification at the ischial level. Since IPS hypertrophy can mimic a tumor, an MRI is useful for differential diagnosis. 7 Although it is not a diagnostic criterion for VNO disease, the lack of increased levels of inflammatory parameters, that is, leukocytes, CRP, and ESR, supports this hypothesis. 6 8

Conservative treatment is the most frequently recommended option, and it consists in antiinflammatory therapy and rest. It is associated with a favorable clinical evolution and disappearance of complaints in about 2 to 3 weeks. 3 5 8 Imaging resolution is more prolonged and may take from several months to 1 year. 3 When ossification is complete, the ischiopubic junction deformity disappears. It is important to note that radiological findings of delayed IPS fusion in asymptomatic children do not result in an ischiopubic osteochondritis diagnosis. 5

On the other hand, VNO disease knowledge and diagnosis as a benign, self-limited condition, allow a targeted approach, without the emotional burden for the patient and his/her family that may be associated to other conditions.
