# Supplementary material for: Van Neck-Odelberg Disease — A Rare or Underdiagnosed Condition?
Source: Rev Bras Ortop (Sao Paulo). 2022 Jan 20;59(5):e797–800. doi: 10.1055/s-0041-1739402 (PMC11624946; doi:10.1055/s-0041-1739402)
Supplement: Related texts

# Research article: Doença de Van Neck-Odelberg — Entidade rara ou subdiagnosticada?

**Keywords:** claudicação intermitente, criança, osteocondrose

## Abstract

A doença de Van Neck-Odelberg (VNO) é uma osteocondrose rara, que acomete a sincondrose isquiopúbica (SIP). Esta entidade deve ser incluída no diagnóstico diferencial de crianças com claudicação na marcha, dor inguinal e limitação funcional do quadril. Imagiologicamente é caracterizada por hipertrofia assimétrica da SIP. Apresentamos o caso clínico de uma criança de 4 anos, previamente saudável, atendida no serviço de emergência devido a dor inguinal no lado esquerdo e claudicação na marcha com início no mesmo dia. Não havia história prévia de trauma ou alteração dos parâmetros inflamatórios. Foi realizada uma radiografia da bacia e ressonância magnética nuclear (RMN) que evidenciou imagem radiopaca de contornos bem definidos no ramo isquiopúbico esquerdo e edema na SIP. Com diagnóstico provável de doença de VNO, o paciente foi medicado sintomaticamente, com resolução completa ao fim de 10 dias. A claudicação na marcha é um motivo frequente de avaliação médica na população pediátrica. Até os 5 anos de idade, as causas mais comuns são sinovite transitória do quadril, artrite séptica e Doença de Legg-Calvé-Perthes. Na ausência de história de trauma ou clínica sugestiva de infeção deverá ser equacionada a hipótese de doença de VNO. Para o seu diagnóstico, é fundamental a realização de radiografia da bacia, cujo achado mais comum é uma opacificação fusiforme unilateral, no nível do ísquion, podendo ser necessária a realização de RMN. O tratamento recomendado é conservador, com recuperação sintomática em 2 semanas. O conhecimento e o diagnóstico desta entidade permitem uma abordagem direcionada e sem a carga emocional para o paciente e seus familiares que outras patologias poderiam transmitir.

## Introdução

As osteocondroses, também denominadas por alguns autores como osteocondrites, resultam de alterações da ossificação endocondral, no nível dos núcleos ósseos em desenvolvimento nas epífises e apófises. 1 2 A sua etiologia permanece desconhecida; contudo, alterações vasculares e microtraumáticas parecem estar na sua origem. 2 Predominam na infância e na adolescência, estando associadas a surtos de crescimento e alterações hormonais. As osteocondroses mais frequentes são a doença de Köhler, doença de Freiberg, doença de Sever e doença de Osgood-Schlatter, sendo a osteocondrite isquiopúbica ou doença de Van Neck-Odelberg (VNO) uma osteocondrose raramente diagnosticada. 1

A osteocondrite isquiopúbica foi descrita inicialmente em 1923 por Van Neck, sendo depois complementada em 1924 por Odelberg, sendo hoje conhecida como doença de Van Neck-Odelberg. 3 4 É uma patologia benigna e auto-limitada, que se caracteriza pela evidência imagiológica de hipertrofia assimétrica da sincondrose isquiopúbica (SIP), associada a claudicação na marcha, dor inguinal unilateral e limitação funcional do quadril. 3 4 Os diagnósticos diferenciais a considerar são a fratura de estresse, patologia infeciosa, nomeadamente osteomielite, ou neoplásica, sendo necessária, em alguns casos, a realização de ressonância magnética nuclear (RMN). 3 5 6

## Relato do Caso

Uma criança do sexo feminino, de 4 anos, previamente saudável, foi atendida no serviço de emergência devido a um quadro com 24 horas de evolução de claudicação na marcha e dor na região inguinal esquerda, de agravamento progressivo. Sem febre, sintomatologia constitucional ou sinais inflamatórios locais e sem história prévia de trauma ou intercorrência infeciosa recente. Ao exame objetivo, destacava-se dor na mobilização do quadril, sem sinais inflamatórios locais.

Foi realizado um estudo analítico, com hemoleucograma, velocidade de sedimentação (VS) e proteína C-reativa (PCR) sem alterações de relevo. A radiografia da bacia evidenciou imagem radiopaca, com cerca de 1.8 × 1.4 cm, de contornos bem definidos, no nível do ramo isquiopúbico esquerdo ( Figs. 1 e 2 ). Para esclarecimento etiológico, foi realizada RMN da bacia, que revelou presença de edema a nível da sincondrose isquiopúbica esquerda, sem alterações dos tecidos moles envolventes ( Fig. 3 ).

**Fig. 1:** Radiografia da bacia (incidência ântero-posterior): imagem radiopaca no nível do ramo isquiopúbico esquerdo, de contornos bem-definidos.

**Fig. 2:** Radiografia da bacia (outlet).

**Fig. 3:** Ressonância magnética da bacia: edema a nível da sincondrose isquiopúbica esquerda.

Excluídas as hipóteses de etiologia infeciosa ou tumoral, foi presumido diagnóstico de doença de VNO, e a paciente foi medicada sintomaticamente, com anti-inflamatório oral e indicação para repouso. Dez dias após, ela foi reavaliada clinicamente, encontrando-se sem queixas.

A radiografia da bacia foi repetida seis meses depois do episódio inicial, verificando-se remodelação completa da área de lesão ( Fig. 4 ). Não houve ocorrência de novos episódios.

**Fig. 4:** Radiografia da bacia (incidência ântero-posterior): resolução imagiológica completa.

## Discussão

A claudicação na marcha é uma queixa frequente na população pediátrica, sendo o seu diagnóstico diferencial muito desafiante, atendendo a múltiplas etiologias possíveis, variáveis de acordo com a faixa etária considerada. Em crianças que apresentam dor unilateral na região inguinal, associada à limitação da mobilidade do quadril, sem febre, história de trauma ou infeções recentes, é importante que seja equacionada a hipótese de doença de VNO. 6

Esta patologia resulta do processo de ossificação assimétrico da SIP, que, habitualmente, tem início na infância e término antes da adolescência. 5 Na maioria dos casos, ela não causa sintomas; contudo, em algumas crianças pode surgir dor na região inguinal ou glútea, bem como limitação da mobilidade articular.

Normalmente, a doença de VNO é mais frequente no membro inferior não dominante, na maioria dos casos o esquerdo, como no caso que apresentamos. Alguns autores explicam este fato pelo peso corporal ser principalmente suportado pelo membro inferior não dominante, comparativamente com o contralateral, que é utilizado preferencialmente para executar os movimentos. 3 5 Este desequilíbrio de forças poderia causar o atraso do encerramento da sincondrose isquiopúbica no lado não dominante, e este, por sua vez, mediante estresse mecânico, tornar-se-ia doloroso. 3 5

A osteocondrite de VNO é um diagnóstico de exclusão em crianças com quadro de claudicação na marcha. 6 Para o seu diagnóstico, é fundamental a realização de radiografia da bacia, cujo achado mais comum consiste na presença de opacificação fusiforme unilateral no nível do ísquion. Atendendo ao aspeto pseudo-tumoral que a hipertrofia da SIP pode assumir, pode ser necessária a realização de RMN para diagnóstico diferencial. 7 Apesar de não constituir critério de diagnóstico de doença de VNO, a ausência de elevação analítica de parâmetros inflamatórios, nomeadamente leucócitos, PCR e VS, apoia esta hipótese. 6 8

O tratamento conservador, com terapêutica anti-inflamatória e repouso, é o mais recomendado, estando associado à evolução clínica favorável e ao desaparecimento das queixas em cerca de 2 a 3 semanas. 3 5 8 A resolução imagiológica é mais prolongada, podendo levar de meses a 1 ano. 3 Quando se completa a ossificação, a deformidade desaparece da junção isquiopúbica. É importante salientar que achados radiológicos com atraso da fusão da SIP em crianças assintomáticas não levam ao diagnóstico de osteocondrite isquiopúbica, constituindo um processo fisiológico normal. 5

Por outro lado, o conhecimento e diagnóstico da doença de Van Neck-Odelburg, de natureza benigna e auto-limitada, permitem uma abordagem direcionada e sem a carga emocional para o doente e seus familiares que outras patologias poderiam transmitir.
